# Ultrastructural Changes Caused by Snf7 RNAi in Larval Enterocytes of Western Corn Rootworm (*Diabrotica virgifera virgifera* Le Conte)

**DOI:** 10.1371/journal.pone.0083985

**Published:** 2014-01-07

**Authors:** Juraj Koči, Parthasarathy Ramaseshadri, Renata Bolognesi, Gerrit Segers, Ronald Flannagan, Yoonseong Park

**Affiliations:** 1 Department of Entomology, Kansas State University, Manhattan, Kansas, United States of America; 2 Department of Biotechnology, Monsanto Company, Chesterfield, Missouri, United States of America; St. Georges University of London, United Kingdom

## Abstract

The high sensitivity to oral RNA interference (RNAi) of western corn rootworm (WCR, *Diabrotica virgifera virgifera* Le Conte) provides a novel tool for pest control. Previous studies have shown that RNAi of DvSnf7, an essential cellular component of endosomal sorting complex required for transport (ESCRT), caused deficiencies in protein de-ubiquitination and autophagy, leading to WCR death. Here we investigated the detailed mechanism leading to larval death by analyzing the ultrastructural changes in midgut enterocytes of WCR treated with double-stranded RNA (ds-DvSnf7). The progressive phases of pathological symptoms caused by DvSnf7-RNAi in enterocytes include: 1) the appearance of irregularly shaped macroautophagic complexes consisting of relatively large lysosomes and multi-lamellar bodies, indicative of failure in autolysosome formation; 2) cell sloughing and loss of apical microvilli, and eventually, 3) massive loss of cellular contents indicating loss of membrane integrity. These data suggest that the critical functions of Snf7 in insect midgut cells demonstrated by the ultrastructural changes in DvSnf7 larval enterocytes underlies the conserved essential function of the ESCRT pathway in autophagy and membrane stability in other organisms.

## Introduction

Western Corn Rootworm *Diabrotica virgifera virgifera* (WCR) is the most important insect pest of maize in North America, and recently also has become a problem in Europe [1]. Genetically modified (GM) maize hybrids expressing *Bacillus thuringiensis* (Bt) Cry toxins have been widely implemented in North America and have largely replaced the use of chemical insecticides for WCR control [2].

Oral RNA interference (RNAi) is a potential next-generation biotechnology that can be used to suppress a target gene of a pest insect thus offering a new approach for rootworm control [3]-[5]. GM corn containing WCR double-stranded RNA (dsRNA) targeting V-ATPase A has been shown to be highly efficient in protecting corn plants from WCR root damage [6]. Additionally, Bolognesi et al [7] showed that dsRNA targeting DvSnf7 (ds-DvSnf7) in WCR was even more toxic than vATPase with an LC_50_ of 4.3 ng dsRNA per mL diet, demonstrating the potential of DvSnf7 as a RNAi target for WCR control.

The Snf7 protein (Snf7/vps32 in yeast and vps32 in nematodes) belongs to a family of proteins with an evolutionary conserved function and is part of the endosomal sorting complex required for transport-III (ESCRT-III). Multiple functions for Snf7 include: 1) autophagy [8]-[11], 2) interaction with de-ubiquitinating enzyme (DUB) to recruit DUB to the ESCRT pathway [12], 3) intraluminal vesicle (ILV) formation in multi-vesicular bodies (MVB) [13], [14], and 4) cytokinesis [15]. In *D. melanogaster*, Snf7 suppression is associated with endosomal cargo sorting [16]. Although the function of the Snf7 protein is conserved, we have demonstrated that suppression of the DvSnf7 gene in WCR via ds-DvSnf7 is sequence specific, and restricted to insects in the Galerucinae subfamily in Coleoptera [17].

Targeting Snf7 with ds-DvSnf7 disrupts a vital cellular function that leads to WCR stunting after five days [7]. Ramaseshadri et al. [18] demonstrated that DvSnf7 RNAi caused the accumulation of ubiquitinated proteins and impairment of autophagic processes in midgut and fat body tissues of dsRNA-fed WCR larvae. However, characterization of the actual cause of autophagy impairment in WCR by DvSnf7 RNAi requires investigation at the subcellular level. In this study, we used electron microscopy (EM) to observe ultrastructural changes in the midgut enterocytes of WCR larvae following exposure to ds-DvSnf7. Accumulation of macroautophagic complexes containing relatively large lysosomes and multi-lamellar bodies in WCR by DvSnf7 RNAi indicated failure of fusion of these organelles to form autolysosomes, leading to impairment of autophagy. In addition, closer observation of enterocytes during the progressive stages of DvSnf7 RNAi by EM analysis indicated loss of membrane integrity of enterocytes. Our data suggest that the essential functions of Snf7 include regulation of autophagy as well as membrane stability in insect midguts.

## Materials and Methods

### Insects

Western corn rootworm (WCR) larvae were obtained from Crop Characteristics (Farmington, MN) and reared at Monsanto Company (Chesterfield, MO). Second-instar larvae were fed an artificial diet containing ds-DvSnf7 or ds-GFP (1000 ng dsRNA/mL diet) or without dsRNA following the protocols described [6]. The above concentration of ds-DvSnf7 was used to demonstrate successfully the mode of action of DvSnf7 in WCR second instar larvae [17]. Treated larvae were maintained at room temp (RT) until sampling on day 5.

### Sample preparation and transmission electron microscopy (TEM)

Whole larvae were briefly washed in deionized H_2_O, submerged in fixative (2% paraformaldehyde/2% glutaraldehyde in 0.1 M cacodylate buffer, pH 7.2), incubated under vacuum (620 mm Hg) for 4 hours, and then further fixed overnight (14 hours) without vacuum at room temperature (RT). When sampling specifically for regions in the 3^rd^ to 4^th^ abdominal segments pertaining to the midgut region, an additional incubation with fixative for 24 hours was performed after samples were cut into smaller pieces along their lengths. All fixed samples were washed (3×30 min) and then incubated overnight at 4°C in 0.1 M cacodylate buffer pH 7.2 (wash buffer) and refixed in 1% osmium tetroxide in 0.1 M cacodylate buffer pH 7.2 (2 hours, RT). The refixed samples were washed 3 times for 1 hour each and dehydrated in ethanol series (50-100%), 3×20 min each. Samples were stored for 2 days at 4°C in 100% ethanol. Spurr's low-viscosity resin (Electron microscopy sciences, Hatfield, PA) was used for infiltration and embedding. Dehydrated samples from the posterior larval abdomen were resin infiltrated at RT as follows: 30 min in 100% propylene oxide (PO), 4 hours in PO:resin (2∶1), overnight (14 hours) in PO:resin (1∶1), 1 hour in PO:resin (1∶3), 4 hours in 100% resin under vacuum (680 mm Hg), and an overnight (14 hours) incubation (TEM protocol by Dr. Daniel Boyle, Division of Biology, Kansas State University) [19]. Samples were then embedded in conical BEEM® capsules (Electron microscopy sciences, Hatfield, PA) for cross-sections of the abdominal 3^rd^ or 4^th^ segment and polymerized at 60°C for 2 days. The ultrathin resin sections (∼95 nm) were cut with an ultramicrotome (Reichert Leica) using a diamond knife (Diatome, Switzerland), transferred to copper grids and contrasted with 2% uranyl acetate (20 min, RT, in dark). Sections were observed at 80 kV using a Philips CM-100 transmission electron microscope (Philips, USA). Images were captured with an AMT digital image capturing system at optimal magnification (range of 1,100 to 13,500x), and image processing was performed using Adobe Photoshop CS5. In this study, three individuals were used for each treatment.

## Results and Discussion

Previous studies have shown that oral RNAi for DvSnf7 in WCR efficiently suppresses the target gene DvSnf7 in a systemic manner [7]. DvSnf7 belongs to the ESCRT pathway, which is required for autophagic degradation of both sequestered cytosolic and endocytosed materials [9]-[12]. Cytoplasmic proteins become sequestered by a phagophore forming double-membrane autophagosomes, which can fuse with lysosomes to generate autolysosomes. Ubiquitinated integral membrane proteins are sorted by ESCRTs into intraluminal vesicles of multi-vesicular bodies (MVB) and are sequestered by the fusion of MVB to autophagosomes and lysosomes [20]. In our previous study, the suppression of DvSnf7 in the WCR gut and fat body was shown to impair processes such as the de-ubiquitination of proteins and autophagy, as manifested by the accumulation of ubiquitinated proteins and disruption of acidic lysosome activity respectively [18]. It is speculated that impairment of autophagy in DvSnf7 dsRNA-fed insects could be an indirect effect due to failure of the fusion of autophagic complexes [15]. Although the enterocytic functions of Snf7 have not been reported previously, the general molecular functions of Snf7 described above must be essential for enterocyte homeostasis and integrity for physiology and development. The ultrastructural observations of the ds-DvSnf7-treated larvae in this study suggest that the DvSnf7 RNAi leads to significant damages on enterocytes, impairing their cellular vital functions.

### Accumulation of macroautophagic complexes in dsDvSnf7 midgut enterocytes

**Figure 1:**
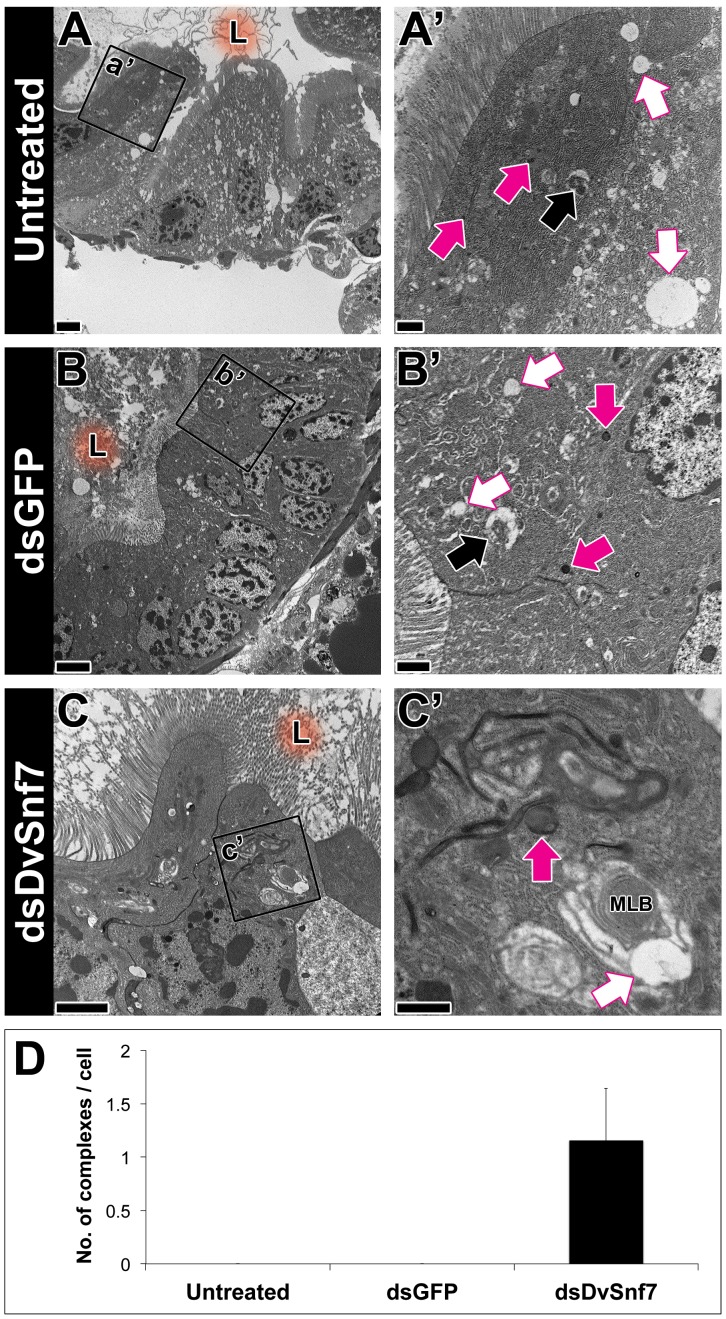
Midgut ultrastructural profiles of untreated, dsGFP-treated and dsDvSnf7-treated western corn rootworm (WCR) second instar larvae fed for 5 days. The letter L with orange glowing background indicates the region of gut lumen, white arrows indicate endosomes, magenta arrows indicate lysosomes, black arrows indicate autolysosomes and MLB is multi-lamellar bodies. C′ is for the magnified view of macroautophagic complex in the midgut of dsDvSnf7-treated individuals. Scale bars  = 4 µm (a, b, c), 1 µm (a′, b′) and 500 nm (c′). D refers to macroautophagic complexes counted in enterocytes of untreated, dsGFP-treated and dsDvSnf7-treated larval midgut. Error bars represent mean ± S.D. of three individuals.

Electron microscopy of control/dsGFP-treated enterocytes revealed the presence of regularly shaped and sized endosomes, lysosomes and autolysosomes (Fig. 1 A, A′, B, B′). The presence of autolysosomes in dsGFP treated enterocytes indicates that the autophagy pathway is functioning properly. In addition, no macroautophagic complexes were observed in the control treatments (Fig. 1 D).

**Figure 2:**
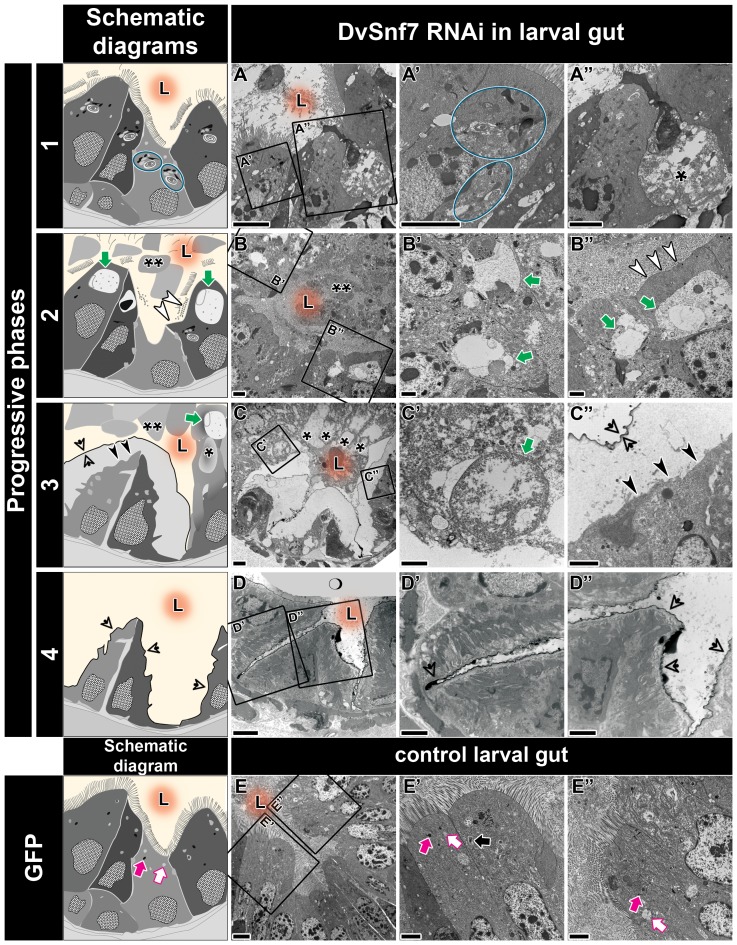
Progression of pathology in the midgut of *dsDvSnf7*-treated WCR larvae. Observed symptoms were summarized in four phases (1 = lightest to 4 = most severe). Highlighted are macroautophagic complexes (blue ellipses) (diagram, A′), gut lumen (L with orange glowing background) filled with cellular debris and loss of microvilli (white arrowheads) (diagrams, B, B″), large vacuoles (diagrams, B′, B″, C′) (green arrows), luminal membrane disintegration (black arrowheads) (diagram, C″) and infolding of the basal membrane (transparent black arrowheads) (diagrams, C″, D′, D″), progressive cell sloughing (asterisks) (diagrams, A″, B, C). Panels E-E″ show midgut of dsGFP-treated control larvae. Highlighted are endosomes (white arrows), lysosomes (magenta arrows) and autolysosomes (black arrow). refers to grey area denoting sectional artifact (cracks in the tissue section). Scale bars  = 4 µm (A-E) and 2 µm (A′-E′ and A″-E″).

In contrast, structures in macroautophagic complexes were observed in ds-DvSnf7 treated enterocytes, which resemble multi-lamellar bodies (Fig. 1 C) [21]. Similar observations were made in mouse neurons treated with mSnf7-2 siRNA, where large numbers of both autophagosome and multi-lamellar bodies were observed [10]. In addition, clusters of these enlarged multi-lamellar bodies were associated with lysosomes (Fig. 1 C′ and Fig. 2 A″), but these structures were never fused together in ds-DVSnf7 treated enterocytes. This suggests that the fusion between the lysosome and multi-lamellar bodies for the formation of an autolysosome is likely the defective step in the ds-DvSnf7 treated enterocytes. Moreover, impaired lysosomal degradation of glycoproteins or glycolipids could be associated with formation of multi-lamellar bodies [22]-[25]. Hence it is possible that accumulation of multi-lamellar bodies in dsSnf7-treated enterocytes could be a consequence of impairment of autophagic degradation of cytosolic materials. Also, Raiborg and Stenmark [26] reported that there is a striking increase in the number of autophagosomes when the ESCRT machinery is inactivated and autolysosomes are rare in ESCRT-depleted cells proposing an apparent connection between ESCRT machinery and autophagy. Consistent with the above observation, absence of autolysosomes and accumulation of macroautophagic complexes (Fig. 1 C) indicates failure in fusion between multi-lamellar bodies and lysosomes in ds-DvSnf7-treated enterocytes.

The observed absence of autolysosomes in ds-DvSnf7 treated enterocytes in this study corroborates our previous findings showing lack of acidic lysosomal activity in ds-DvSnf7-treated enterocytes of WCR [18] and larval fat bodies of *vps32* (Snf7 ortholog) mutant *Drosophila*. Taken together, it is likely that accumulation of macroautophagic complexes due to the lack of fusion with lysosomes and absence of autolysosomes in ds-DvSnf7-treated enterocytes indicates malfunctioning of autophagic processes.

In addition, macroautophagy is a subtype of autophagy, in which the non-specific engulfment of bulk cytoplasm or organelles occurs [27]. Although autophagy is known to be an essential process for cell homeostasis, the impairment of macroautophagy appears to be induced as a consequence of the pathology, such as the aggregation of proteins and lipids [11], [28], [29]. Therefore, the impairment of macroautophagy in the ds-DvSnf7-treated larvae is likely a pathological symptom.

### Progression of the pathological symptoms in ds-DvSnf7 enterocytes

The progression of pathological symptoms in enterocytes from WCR fed ds-DvSnf7 can be summarized into 4 phases. In the first phase, cell sloughing and apical swelling were observed in midgut enterocytes with the frequent occurrence of macroautophagic complexes (Fig. 2 A, A′, A″). In the second phase, the loss of microvilli at the apical surface and cell sloughing resulted in the lumen being filled with cellular debris (Fig. 2 B, B′, B″). In this phase, we could observe progression of macroautophagic complex to large vacuoles (Fig. 2 B′). The large vacuoles indicate the product of fusions among macroautophagic complex and endosomal components. Although the roles of autophagy and large vacuoles in the enterocytes of metamorphic insects have been previously described [30], it remains unclear whether the large vacuoles in ds-DvSnf7 enterocytes are equivalent to the previously described degenerative process of the cells. The third phase of pathology progression displayed extensive apical swelling of the cells and massive cell sloughing toward the lumen (Fig. 2 C). At this phase, the apical regions of enterocytes are filled with electron-lucent space, whereas an electron-dense membranous layer separates the cell and luminal space (Fig. 2 C, C′, C″). The last and most severe pathological phase is characterized by the loss of the cellular contents. Paired or single enterocytes attached to the basal layer were likely separated by the infoldings of the electron-dense layer (Fig. 2 D, D′, D″). At this phase, the gut was approximately 2-fold smaller in diameter (40 µm), compared to the gut size in the third phase (80 µm).

One of the most important functions of Snf7 is to recruit the de-ubiquitinating enzyme (DUB) for recycling ubiquitinated proteins [10]. The membrane receptors for signal transduction between neighboring cells, such as Notch and EGFR (epidermal growth factor receptor), are well known targets of the de-ubiquitination mediated through ESCRTs [10], [12], [16], [18]. As a consequence of defects in recruiting DUB in the ds-DvSnf7-treated larvae, reduced recycling of membrane signaling proteins may have led to loss of the apical membrane and cell-cell junction integrity. In addition, Vaccari et al. [16] observed the presence of actin cytoskeleton defects and fragmented plasma membrane suggesting the loss of plasma membrane integrity in ESCRT-III (Vps2) mutant germ cell. These data suggest that ESCRT has a role in stabilizing the plasma membrane and this novel function of ESCRT might operate in WCR enterocytes.

## Conclusion

Taken together, the major ultrastructural pathological effects in DvSnf7 RNAi enterocytes were macroautophagic complexes that are likely the consequence of the defective fusion between the multi-lamellar bodies and lysosomes. Through EM analysis we were able to visualize the possible essential function of Snf7 in the fusion of autophagic complexes, which is essential for autophagy. The loss of cell membrane integrity and cell-cell junctions points to an impairment in the process of de-ubiquitination of membrane signaling proteins. The severe cellular defects in the enterocytes described in this study are likely the major cause of death at the organismal level after ds-DvSnf7 treatment of WCR. The lethality caused by oral DvSnf7RNAi in WCR suggests malfunctioning of cellular processes such as autophagy and membrane stability.
